# Efficacy of Bezlotoxumab Against Clostridioides difficile Infection: A Case-Series Study at a University Hospital in Japan and Literature Review

**DOI:** 10.7759/cureus.42779

**Published:** 2023-08-01

**Authors:** Nobuaki Mori, Jun Hirai, Nobuhiro Asai, Hiroshige Mikamo

**Affiliations:** 1 Department of Clinical Infectious Diseases, Aichi Medical University, Aichi, JPN

**Keywords:** retrospective cohort study, human monoclonal antibody, recurrence, bezlotoxumab, clostridioides difficile infection

## Abstract

Background

*Clostridioides difficile* infection (CDI) recurrence is a public health concern as well as a health economic burden. Bezlotoxumab treatment is one way to prevent recurrence; however, its clinical results have not been reported in Japan. Therefore, we investigated the efficacy and safety of bezlotoxumab in patients with CDI at a university hospital in Japan and compared them with previously reported findings.

Methodology

We retrospectively examined all patients with some risk factors for recurrent CDI who received bezlotoxumab at the discretion of physicians at the Aichi Medical University Hospital, Aichi, Japan, between July 2018 and July 2022. The primary outcome was the three-month CDI recurrence rate. The secondary outcomes were an initial clinical cure and the six-month CDI recurrence rate. The safety of the administration was also assessed.

Results

A total of nine patients who received bezlotoxumab were included during the study period. The rate of CDI recurrence within three months was 28.5% (2/9). Two patients died due to other causes before their diarrhea improved. None of the patients experienced CDI recurrence between three and six months after the initial clinical cure of the baseline episode. Patients showed good tolerability to bezlotoxumab with no adverse effects. Two patients with a single episode of CDI recurrence before bezlotoxumab administration showed no recurrence.

Conclusions

In this Japanese case-series study, the efficacy of bezlotoxumab in preventing CDI recurrence in elderly patients with CDI and multiple underlying diseases was inferior to that reported in previous studies that analyzed real-world data. It is possible that bezlotoxumab may not be fully effective in elderly patients with CDI.

## Introduction

*Clostridioides difficile *infection (CDI) is the leading cause of healthcare-associated diarrhea, and its recurrence is problematic because bacterial spores can survive in the intestinal tract after treatment. Up to 25% of patients experience recurrent CDI within 30 days of treatment [1]. Once patients experience a single recurrence, they are at a significantly increased risk of subsequent recurrences.

Bezlotoxumab, a human monoclonal antibody against toxin B of *C. difficile*, was approved by the US Food and Drug Administration in October 2016 and by the Japanese Ministry of Health, Labour, and Welfare in September 2017. Bezlotoxumab reduces recurrent CDI in adults above 18 years of age who are at a high risk of and receive antibacterial treatment for recurrent CDI. However, using bezlotoxumab in real-world clinical practice is challenging due to some barriers, including reimbursement issues for inpatients, the need for prior authorization through insurance coverage, and scheduling infusions in outpatient settings. Therefore, data from real-world settings since the launch of bezlotoxumab, particularly in Japan, are limited.

Here, we investigated the efficacy of bezlotoxumab in patients with CDI at a university hospital in Japan and reviewed previously published studies analyzing real-world data.

## Materials and methods

This retrospective cohort study was conducted at the Aichi Medical University Hospital, a 900-bed tertiary-care hospital in Aichi, Japan. All patients who received bezlotoxumab between July 2018 and July 2022 were included. The following data were collected from the electronic medical records: age, sex, principal illness on admission, underlying disease, type and number of antibiotics administered two months before CDI diagnosis, use of acid suppressants, use of immunosuppressants and anti-cancer drugs, history of abdominal surgery, nutrition pathway (disruption of feeding, parenteral nutrition, and enteral feeding), history of CDI, use and type of prescribed probiotics and concomitant standard of care (SoC) antibiotics for CDI, and reasons for using bezlotoxumab. In addition, we evaluated the following risk factors for recurrence based on those used in the MODIFY I/II studies: (i) ≥65 years old; (ii) one or more CDI episodes in the past six months; (iii) immunocompromised; (iv) severe CDI (defined as Zar score ≥2; scores range from 1 to 8, with higher scores indicating more severe infection [2]); and (v) CDI caused by a highly virulent strain (polymerase chain reaction (PCR) ribotype 027, 078, or 244 strain). However, we could not analyze the PCR ribotype and excluded it from the evaluation. In addition, severity was evaluated based on the Society for Healthcare Epidemiology of America (SHEA)/Infectious Diseases Society of America (IDSA) guidelines [3] and MN criteria [4]. The SHEA/IDSA guidelines specify severe CDI as a white blood cell (WBC) count >15,000 cells/mL or serum creatine level ≥1.5 mg/dL. The MN criteria, proposed as the Japanese CDI severity scoring system, are based on the following nine categorical variables: age, abdominal pain/distention, body temperature, diarrhea counts, hematochezia, WBC counts, estimated glomerular filtration rate, serum albumin, and image findings; each variable is scored using a three-point scale: ≤4 points, mild CDI; 5-9 points, moderate CDI; 10-13 points, severe CDI; and ≥14 points, super-severe CDI [4].

*C. difficile* stool testing and culture can be ordered by physicians in cases of three or more episodes of diarrhea (Bristol Stool Scale score, 5-7) and one additional symptom of CDI. CDI was diagnosed based on the presence of any gastrointestinal symptoms accompanied by clinical suspicion of CDI and a positive result for* C. difficile* toxins according to a rapid immunoenzyme test for glutamate dehydrogenase (GDH) and a toxin assay (C. Diff Quik Chek COMPLETE kit; TechLab, Blacksburg, VA, USA, or GE Test Immunochromato-CD GDH/TOX; Nissui Pharmaceutical Co., Ltd, Tokyo, Japan) or toxin B gene using the GeneXpart *C. difficile* PCR assay (“Cepheid”) (Beckman Coulter, Inc., Tokyo, Japan). The C. Diff Quik Chek COMPLETE kit was used between July 2018 and March 2022, while the GE Test Immunochromato-CD GDH/TOX was used from April 2022. The reason for administering bezlotoxumab was based on the insurance benefit indication in Japan: immunocompromised, severe CDI, CDI caused by a highly virulent strain (PCR ribotype 027, 078, or 244), three or more previous CDI episodes, and when the patient was judged to be at high risk of serious disease or recurrence for other reasons.

The primary outcome was the three-month recurrence rate of CDI, defined as a new episode of CDI after the initial clinical cure of the baseline episode. The secondary outcomes were the initial clinical cure, defined as no diarrhea for two consecutive days after SoC, and CDI recurrence within six months, defined as a new episode of CDI after the initial clinical cure of the baseline episode. The safety of bezlotoxumab administration was also assessed through a retrospective review of medical records. Bezlotoxumab has been reported to induce heart failure and infusion-related reactions.

## Results

During the study period, nine patients received bezlotoxumab treatment. Six patients received bezlotoxumab within the SoC period, and the remaining three patients received it on days 20, 28, and 31 after starting the SoC for refractory CDI because their diarrhea did not improve with SoC. The demographic and baseline characteristics of the study population are presented in Table 1. The median age was 84 years (range = 49-90 years), and five patients (55.6%) were women. Three of the nine patients (33.3%) were outpatients, and the remaining six were hospitalized. Six of the nine patients (66.7%) received gastric antacids, such as proton pump inhibitors and potassium-competitive acid blockers.

**Table 1:** Demographic and baseline characteristics of the study participants. CD: *Clostridioides difficile*; MEPM: meropenem; CLDM: clindamycin; A/S: ampicillin/sulbactam; VCM: vancomycin; T/P: tazobactam/piperacillin; FLCZ: fluconazole; CMZ: cefmetazole; MCFG: micafungin; DAP: daptomycin; MNZ: metronidazole; FDX: fidaxomicin; VGCV: valganciclovir; PPI: proton pump inhibitor; P-CAB: potassium-competitive acid blocker; PSL: prednisolone; MMF: mycophenolate mofetil; SLE: systemic lupus erythematosus; SjS: Sjögren’s syndrome; ITP: idiopathic thrombocytopenic purpura; N/A: not applicable

Patient number	Age	Sex	Setting	Patient characteristics								Severity			Probiotics	Antibiotics during anti-CD drugs	Concomitant anti-CD drugs	Total duration of anti-CD drugs (days)	Days from initiation of SoC to bezlotoxumab, median days	Outcome
				Underlying disease	Administering antibiotics within 2 months	Gastric antacid	Immunosuppressant	Anticancer drug	History of abdominal surgery	Tube feeding	Episodes of Past CD infection	Zar Score	MN criteria score	SHEA/IDSA guideline severity						
1	86	M	Inpatient	Necrotizing fasciitis, benign prostate hyperplasia	MEPM, CLDM, A/S, VCM i.v.	PPI	N/A	N/A	N/A	N/A	0	Severe	10	Severe	Biofermin, miyaBM	MEPM	MNZp.o.	14	3	Recurrence within 3 months
2	49	M	Outpatient	Ulcerative colitis	N/A	N/A	PSL	N/A	N/A	N/A	>3	Unevaluable	Unevaluable	Unevaluable	N/A	N/A	VCM	88	31	No recurrence
3	94	F	Outpatient	Ulcerative colitis, gastric cancer, hypertension	N/A	PPI	N/A	N/A	N/A	N/A	>3	Non-severe	3	Non-severe	N/A	N/A	VCM	28	28	No recurrence
4	50	F	Outpatient	Ulcerative colitis, breast cancer, uterine body cancer	N/A	N/A	N/A	N/A	+	N/A	2	Non-severe	0	Non-severe	miyaBM	N/A	VCM	14	9	No recurrence
5	84	F	Inpatient	Liver cirrhosis, cholangitis	MEPM	PPI	N/A	N/A	N/A	N/A	1	Severe	10	Non-severe	N/A	MEPM	VCM+MNZiv	10	0	Death
6	76	M	Inpatient	Burn, cerebral hemorrhage	MEPM, T/P, FLCZ	PPI	N/A	N/A	N/A	+	2	Severe	9	Severe	N/A	MEPM	MNZp.o.	14	2	Recurrence within 3 months
7	76	M	Inpatient	Lung cancer, cholelithiasis	CMZ, MEPM, MCFG, DAP	PPI	N/A	N/A	N/A	+	1	Severe	10	Severe	miyaBM	MEPM+DAP	MNZ→FDX	24	20	Death
8	88	F	Inpatient	Osteomyelitis (foot), hypertension	DAP, T/P. A/C	N/A	N/A	N/A	N/A	N/A	1	Severe	11	Non-severe	miyaBM	DAP	FDX	10	2	No recurrence
9	86	F	Inpatient	Diabetes mellitus, SLE, SjS, ITP	VGCV	P -CAB	PSL, MMF	N/A	N/A	N/A	1	Non-severe	8	Non-severe	miyaBM	VGCV	FDX	10	8	No recurrence

The frequency of risk factors for recurrent CDI in the patients, based on those of the MODIFY I/II studies, is presented in Table 2. The median number of risk factors for CDI was three. Five cases of severe CDI met the SHEA/IDSA guidelines or MN criteria, with three cases meeting the SHEA/IDSA criteria (Cases 1, 6, and 7), four meeting the MN criteria (Cases 1, 5, 7, and 8), and two meeting both (Cases 1 and 7); for one case (Case 2), blood tests were not performed. Regarding the reasons for administering bezlotoxumab based on the insurance benefit indication, two patients were immunocompromised (22.2%), two had experienced three or more previous CDI episodes (22.2%), five had severe CDI (55.6%), and one had other reasons (the physician judged the patient to be at high risk of serious CDI or recurrence) (11.1%).

**Table 2:** Frequency of the number of recurrent Clostridioides difficile infection (CDI) risk factors. *: Zar score

Patient No.	Recurrent CDI risk factor				Number of risk factors
	≥65 years	One or more CDI episodes in the past 6 months	Immunocompromised	Severe CDI*	
1	◯	-	-	◯	2
2	-	◯	◯	-	2
3	◯	◯	-	-	2
4	-	◯	-	-	1
5	◯	◯	-	◯	3
6	◯	◯	-	◯	3
7	◯	◯	-	◯	3
8	◯	◯	-	◯	3
9	◯	◯	◯	-	3

Regarding antibiotic administration and duration of CDI treatment with bezlotoxumab, two of the nine patients were administered metronidazole orally for 14 days, one was administered vancomycin for 14 days, two were administered fidaxomicin for 10 days, and one was administered metronidazole and vancomycin intravenously for 10 days. Three of the nine patients showed no improvement in their clinical symptoms, including diarrhea, during SoC. Therefore, two patients (Cases 2 and 4) received vancomycin for 28 and 88 days, respectively, until they were confirmed to be negative for the *C. difficile* toxin by the physician. One patient (Case 7) received metronidazole for 10 days, followed by fidaxomicin for 14 days.

Two patients experienced recurrent CDI three months after the initial clinical cure of the baseline episode. Regarding clinical outcomes, diarrhea improved in four patients during SoC, but three patients did not show a clinical cure because of persistent diarrhea due to complications, such as refractory ulcerative colitis and severe pancreatitis. Two patients died due to other causes before diarrhea improved (Cases 5 and 7). Therefore, it was not possible to demonstrate a cure for CDI in this study. The rate of CDI recurrence within three months was 28.5% (2/7). None of the patients presented with CDI recurrence between three and six months after the initial clinical cure of the baseline episode. Consequently, two (28.5%) patients experienced recurrent CDI six months after the initial clinical cure of the baseline episode. Bezlotoxumab was well tolerated, with no adverse effects, including heart failure and infusion-related reactions.

## Discussion

Here, we performed a case-series study on real-world data of bezlotoxumab administration in patients with CDI recurrence risk at a university hospital in Japan. The CDI recurrence rate within three and six months was 28.5%. As in the present study, low bezlotoxumab efficacy in preventing CDI recurrence in elderly patients with multiple underlying diseases is to be expected.

In our study, the rate of CDI recurrence within three months of the initial clinical cure of the baseline episode was 28.5%. The CDI recurrence rate within three months after CDI diagnosis at our hospital during the same period was 15.3% (19/124; data not shown). No significant CDI recurrence prevention effect was observed. In Japan, epidemiological data for the country as a whole have not been published yet, but single- and multicenter studies have reported a CDI recurrence rate of 3.3-30% [5-7]. A retrospective cross-sectional analysis enrolling 320 Japanese Diagnosis-Procedure Combination hospitals between 2012 and 2016 showed that the CDI recurrence rate was 11.5% (1,359/11,823), which is lower than that in other countries. The same study also showed that recurrent CDI is associated with high costs and long hospitalizations, thereby significantly burdening the healthcare system [7]. Other studies have reported CDI recurrence rates exceeding 20% in enrolled patients who underwent solid organ transplants or those with pediatric cancer [6]. Two global phase III studies (MODIFY I/II) demonstrated that the CDI recurrence rate was significantly lower in the bezlotoxumab group than that in the placebo group within 12 weeks of administration [8]. In a sub-analysis of Japanese patients from the MODIFY II study, the CDI recurrence rate was 46% in the placebo group and 21% in the bezlotoxumab group (p = 0.0197) [9]. The efficacy of bezlotoxumab in preventing CDI recurrence was superior to that observed in our study. We reviewed real-world data on bezlotoxumab use for CDI treatment published between 2019 and 2022 (Table 3) [10-17]. Although eight studies were applicable, a study [17] comparing the efficacies of fidaxomicin and bezlotoxumab in preventing CDI recurrence was not included in this literature review because it used the same database as other studies [16]. In various studies, it was pointed out that the CDI recurrence rate within three months under bezlotoxumab treatment was 11-27% [10-16]. Compared with these studies, the CDI recurrence rate using bezlotoxumab in our study was 28.5%, indicating diminished bezlotoxumab efficacy. In this regard, it is possible that as the patients had a background of various underlying diseases and were elderly, with a median age of 84 years, immunosenescence may be a contributing factor. Further studies comparing groups with similar CDI recurrence risks are warranted to validate the efficacy of bezlotoxumab.

**Table 3:** Real-world data on preventing CDI recurrence using bezlotoxumab. N/A: not available; CDI: *Clostridioides difficile* infection; VCM: vancomycin; MNZ: metronidazole; FDX: fidaxomicin; ACG: American College of Gastroenterology; ESCMID: European Society of Clinical Microbiology and Infectious Diseases; SHEA/IDSA: Infectious Diseases Society of America  and the Society for Healthcare Epidemiology of America; IQR: interquartile range

Reference	Nation	Applicable period	Number of patients	Age (year)	Rate of outpatient	Immunocompromised	Severe CDI	Criteria of severe CDI	Concomitant anti-CD drug	Days from the initiation of SoC to bezlotoxumab, median days (range)	Recurrence rate within 3 months
Johnson et al., 2022 [15]	US	2015 to 2019	53	Mean 55	87% (46/53)	77% (41/53)	23%	Zar score	VCM 48, FDX 32, combination MNZ IV 8	19 (12–35)	11% (6/53)
Johnson et al., 2021 [14]	US	January 2015 to November 2019	38	Mean 51	0%	100% (38/38)	13%	Zar score	VCM 33, FDX 13, combination 5	25 (IQR: 13–40)	16% (6/38)
Oksi et al., 2019 [10]	Finland	2017	46	Mean 66	37%(17/46)	61% (28/46)	39%	Zar score	VCM 37, MNZ 9, FDX 7, TGC 2	N/A	27% (12/44)
Hengel RL al., 2020 [11]	US	April 2017 to December 2018	200	Median 70	100%	42% (82/200)	28%	ACG guideline 2013	VCM 76, VCM taperd 61, FDX 60, MNZ 3	11 (2–144)	15.9%(31/195)
Olmedo et al., 2021 [12]	Spain	Aug 2018 to Sep 2019	16	Median 69.5	N/A	87.5% (14/16)	56.3%	ESCMID guideline 2014	VCM14, FDX 2	N/A	21.4% (3/14)
Askar et al., 2022 [13]	US	June 2017 to November 2018	23	Median 63	0%	82.6% (19/23)	N/A	N/A	VCM 16, VCM+MNZiv 5, MNZ 2, FDX 2	N/A	13% (3/23)
Escudero-Sánchez et al., 2020 [16]	Spain	Jul 2018 to Jul 2019	91	Median 71	N/A	61.5% (56/91)	45.1.% (Zar), 38.5% (IDSA)	Zar score and SHEA/IDSA guideline 2013	VCM 40, VCM tapered 32, FDX 9, VCM+MNZ 5, FDX extended 4, FMT 1	N/A	14.2% (13/91)
-	Japan (Our study)	July 2018 to July 2022	9	Median 84	33.3% (3/9)	22.2% (2/9)	44.4% (MN criteria), 55.5% (Zar), 33.3% (SHEA/IDSA)	MN criteria, Zar score, and SHEA/IDSA guideline 2021	VCM 3, MNZ 2, FDX 2, VCM+MNZ1, MNZ→FDX 1	8 (0–31)	28.5% (2/7)

Regarding the timing of administration, in this study, bezlotoxumab was administered to six patients during the SoC period and three patients who had persistent diarrhea on days 20, 28, and 31 after the start of SoC. The effect of bezlotoxumab administration timing on the treatment outcomes is unclear. A sub-analysis of data from the MODIFY I/II trials evaluated the effect of bezlotoxumab on CDI recurrence prevention on 0-2, 3-4, and ≥5 days after CDI treatment [18]. The results showed that the CDI recurrence rate was lower in the bezlotoxumab treatment groups than that in the placebo group at 19.3, 20.4, and 22.8% at 0-2, 3-4, and ≥5 days, respectively, regardless of bezlotoxumab administration timing. Although no statistical analysis was performed, the same study showed a trend toward a lower relapse rate with earlier administration. However, real-world data have shown that bezlotoxumab tends to be administered much later after CDI onset [11,14,15]. In contrast, a risk analysis conducted by Hengel et al. revealed that having ≥2 CDI recurrences before bezlotoxumab treatment was a significant predictor of subsequent CDI recurrence [11]. In our study, patients with a single episode of CDI recurrence before bezlotoxumab administration showed no recurrence after administration, except those who died of other causes. These data suggest that bezlotoxumab administration during early CDI episodes may improve patient outcomes.

The duration of the efficacy of bezlotoxumab in preventing long-term CDI recurrence remains unknown. In this study, we followed up patients for up to six months after the initial clinical cure of the baseline episode. No CDI recurrence was observed between three and six months after the initial clinical cure of the baseline episode. A nine-month extension cohort of the MODIFY II trial showed toxigenic *C. difficile* colonization rates of 16-32% within 12 months of bezlotoxumab infusion but no cases of CDI recurrence were observed during the nine-month extension period [19]. An analysis of the pharmacokinetics and pharmacodynamics of bezlotoxumab showed that its elimination half-life was approximately 19 days, and measurable concentrations of bezlotoxumab could be detected in the serum during the first 12 weeks to up to 24 weeks after treatment based on limited sampling in some participants [20]. Thus, the effect of bezlotoxumab on CDI recurrence observed in the extended cohort study may be due to a prophylactic effect against CDI recurrence rather than a delay in the development of CDI recurrence after the decline of antibody levels. However, data collected over a longer period are needed to confirm the efficacy of bezlotoxumab in preventing recurrent CDI.

This study had several limitations. Our study was performed at only one institution and had a limited sample size, thus limiting the generalizability of our findings. Moreover, the effect of ribotype 027 on the results of this study is unclear because we could not perform the ribotyping of the isolated *C. difficile*. However, ribotype 027 has rarely been isolated in Japan.

## Conclusions

The results of this single-center, Japanese case-series study demonstrated that the effectiveness of bezlotoxumab in preventing CDI recurrence was lower than that reported in previous studies analyzing real-world data. However, other episodes of CDI recurrence were not observed during the six-month follow-up. Further studies are needed to determine the appropriate timing and subjects for bezlotoxumab administration to prevent CDI recurrence.
